# Effect of diode laser on peri-implantitis by monitoring gingival crevicular fluid levels

**DOI:** 10.6026/973206300210096

**Published:** 2025-01-31

**Authors:** Saikiran Bahadur, Aseem Sharma, Omar Basheer Altaher Mohammed, Liya Zacharias Vithayathil, Sonali Mahajan, Sajida Aboobacker, Nubesh Khan Syed, Nishita Gogoi, Tanushree Sharma

**Affiliations:** 1 Departments of Prosthodontics, Dental Director, Springfield Dental, 4 Matthew drive, Suffield Connecticut, USA; 2 Department of Orthodontics and Dentofacial Orthopedics, HIDS Paonta Sahib, Himachal Pradesh, India; 3 Department of Oral and Maxillofacial Surgery, Sebha Dental Faculty, Sebha University, Lybia, North Africa; 4 Department of Prosthodontics, PSM College of Dental Sciences, Kerala, India; 5 Department of Prosthodontics, Government Dental College and Hospital, Aurangabad, Maharashtra, India; 6 Specialist Periodontist, Veema Dental Center, Dilaymiyah, Al Qassim, Saudi Arabia; 7 Department of Periodontology and Implant Dentistry, College of Dentistry, Qassim University, Buraydah, Qassim, Kingdom of Saudi Arabia; 8 Intern, Kalinga Institute of Dental Sciences, Bhubaneswar, Odisha, India; 9 Department of Dentistry, Shri Mata Vaishno Devi Institute of Medical Excellence (Medical College and Associated Hospital), (SMVDNSH), Kakryal, Jammu and Kashmir, India

**Keywords:** Crevicular fluid, dental implant, diode laser, interleukin, peri-implantitis

## Abstract

Untreated peri-implantitis results into implant failure. Therefore, it is of interest to assess the effect of diode laser on peri-implantitis and levels of crevicular fluid biomarkers. Hence, this study involved 20 participants, with a total of 24 dental implants exhibiting peri-implantitis on the laser group (n = 10) and the control group (n = 10). Clinical parameters around the implants were evaluated and samples of peri-implant crevicular fluid (PICF) were collected at baseline, as well as at 3-month and 6-month follow-up intervals. These groups exhibited significant differences in terms of periodontal parameters and IL-1β levels in PICF at the 3rd and 6th month follow-up visits showing diode lasers as a dependable tool.

## Background:

Dental implants are commonly used to restore the missing tooth. The success of implant is depending on local and systemic factors (systemic health of the patient). Local factors such as implant type, bone quality, periodontal health, absence of peri-implantitis and tobacco habits. Progressive bone Loss in the supporting peri-implant region is a defining feature of peri-implantitis [1]. Peri-implant mucositis can proceed to peri-implantitis if left untreated, which can result in the loss of dental implants [2]. Peri-implant infections show similar etiological variables like periodontal disorders [3]. The 2017 World Periodontology Workshop Consensus Report categorized peri-implant disorders as periodontal diseases. Peri-implant pockets that are at least 4 mm deep, bleeding and/or purulent discharge, an inflammatory disease linked to varying degrees of bone loss surrounding a dental implant are some of the symptoms of peri-implantitis (PI) [4]. Peri-implantitis prevalence ranged from 1.1% to 85.0%, according to Dreyer *et al.* [5]. Peri-implantitis is largely caused by poor plaque control, hence the removal of the biofilm from the implant site and a comprehensive mechanical debridement are necessary for peri-implant infection control [4]. Peri-implantitis frequently manifests as edema, redness, mucosal enlargement, deepening of the pocket, radiographic marginal bone, bleeding on probing (BOP) with or without suppuration, loss which are clinical indicators of inflammation. Interleukin (IL)-1β is one of the cytokines that is crucial in peri-implant disorders since it controls collagenase activity in inflammation and wound healing. Alveolar bone resorption and the inflammatory response are both significantly influenced by IL-1β [2, 6]. Samples of saliva or gingival crevicular fluid (GCF) can be used to quantitatively evaluate biomarkers. The precision of determining the volume and content of GCF is impacted when samples are contaminated by blood, saliva, or dental plaque [7]. The stabilization of the bone attachment and the resolution of peri-implant soft tissue inflammation must be the goals of peri-implantitis treatment [8]. Several therapeutic approaches have been proposed in the literature for the management of peri-implantitis, which includes the use of anti-infective agents, respective (the most common epilepsy surgery) or regenerative surgical treatments and combined treatments, mechanical debridement, oral hygiene instructions, antibiotic medicine, chemical modalities, laser application and air-abrasives [9, 10]. Considering the public health issue regarding the rise in antibiotic resistance and local application of antimicrobials can be beneficial to conventional periodontal treatment [11]. In individuals with extensive periodontal pockets, sub gingival instrumentation-with or without additional therapies-has been proposed to be inadequate [12]. Decontamination of the implant site is crucial and traditional non-surgical therapy methods for peri-implant diseases showed low predictability. Numerous supplementary instruments, including photodynamic treatment, have been suggested and examined in preclinical and clinical research [3]. Based on photo-bio-modulation (low-level lasers (LLL) or light-emitting diodes (LEDs)) have been extensively used as an adjuvant therapy for the treatment of periodontitis within the visible red or near-infrared (NIR) range of the spectrum (600 to 700 nm and 780 to 1100 nm) [13]. Due to their inability to interact with titanium or coated materials, diode laser (photodynamic) therapies have demonstrated efficacy in cleaning implant surfaces and bio stimulating peri-implant tissues without producing complications in the surrounding tissues [3, 6]. Therefore, it is of interest to assess the efficiency of diode laser technique for non-surgical management of peri-implantitis.

## Materials and Methods:

After receiving approval from the relevant authorities and obtaining informed consent from each participant, this cross-sectional research was done. The study's inclusion criteria included having at least one dental implant with a diagnosis of peri-implantitis and not having any systemic chronic conditions or medications known to affect periodontal health. Patients receiving any kind of NSAID (Non-steroidal anti-inflammatory drugs), probiotic, or antibiotic treatment were excluded. Supra-gingival cleaning and oral prophylaxes were conducted two weeks prior to the study. A Total of 20 patients diagnosed with peri-implantitis were randomly assigned to either the laser (LG) or control (CG) groups. Each group was assigned 10 samples containing 12 implants. In the control group, fallowing local anaesthesia, titanium curettes were used to remove hard deposits around each dental implant. Stainless steel curettes (Hu-Friedy, Chicago, IL) were used to curette the inflamed peri-implant soft-tissue wall of the pocket. Ultimately, the sulcus was irrigated with sterilized saline solution and the area was sutured. Excision of inflammatory tissue and calcified deposits was performed in the laser group similar to control group. Subsequently, adjunctive diode laser therapy (Epic, Biolase, Irvine, CA) was administered in a continuous mode ata wavelength of 940 nm, with a power output of 0.80 W and an energy level of 0.80 J/s, utilizing an optical fiber tip with a diameter of 300 μm positioned at the most apical region of the inner peri-implant pocket, parallel to the dental implant surface. The laser point was methodically moved in an apico-coronal and mesio-distal manner, wiped consistently with sterile gauze during the procedure to monitor blood coagulation formation. All the procedure was conducted by a single qualified investigator. The periodontal was employed to evaluate clinical parameters, including probing depth (PD), clinical attachment level (CAL), bleeding on probing (BOP), plaque index (PI) and gingival index (GI), at baseline, 3 and 6 months post-treatment, at four sites per dental implant. The peri-implant crevicular fluid (PICF) samples were collected from four sites per dental implant (disto-buccal, mesio-buccal, mid-palatal and mid-buccal/lingual regions) using sterile paper strips (Periopaper, Ora-Flow, Amityville, NY, USA) at baseline, 3 and 6 months post-treatments. Subsequently, it was subjected to ELISA analysis for the quantification of biomarkers (IL-1β interleukins) after being stored at - 82°C in a single Eppendorf tube. The ANOVA test was employed to statistically analyze the data obtained using SPSS software (SPSS v-24, IBM Corp, NY and USA). The statistical significance level was set at p < 0.05.

## Results and Discussion:

Table 1 shows improvement in PD, PI, GI and decrease in IL-1β interleukin biomarker in both the groups from baseline to 3 months to 6 months of follow-up. There was better improvement in laser group compared to control group. The clinical changes in peri-implant tissues and the analysis of biomarker levels involved in the pathogenesis of peri-implantitis, both pre and post-treatment interventions, could be used to evaluate the process of peri-implantitis development and the inherent ambiguity in its treatment [2]. The diagnosis of peri-implantitis is contingent upon the presence of inflammatory mediators in the peri-implant crevicular fluid (PICF) [6]. This investigation was intended to ascertain whether diode laser treatment has any beneficial effects in the treatment of peri-implantitis in comparison to the control non-laser group, both clinically and biochemically. In this study, both groups exhibited substantial decrease in clinical periodontal parameters from the baseline to the third and sixth months (Table 1). In both groups, there was a substantial decrease in PICF- IL levels over time. The diode laser was administered in conjunction with mechanical therapy, which resulted in a more significant reduction in bleeding on probing. In our investigation, the IL-1β level was assessed as a biomarker. In our investigation, a diode laser was employed to treat peri-implantitis due to the potential effect of diode laser system for implant decontamination. Erduran *et al.* conducted a similar assessment of the immunological and clinical efficacy of diode laser therapy as an alternative to non-surgical mechanical therapy in peri-implantitis cases. They concluded that diode laser seems to be a dependable tool as an adjunct for supporting the nonsurgical mechanical treatment [2]. Altindal *et al.* evaluated the efficacy of a 940-nm diode laser for the non-surgical treatment of PI. They concluded that the diode laser demonstrated betterment in clinical parameters in the peri-implant tissue [6]. Aimetti *et al.* demonstrated that diode laser treatment did not offer a statistically considerable clinical advantage in the management of peri-implant inflammation at three months when contrasted with nonsurgical mechanical therapy alone [14]. The Results of adjunctive diode laser application in the management of non-surgical therapy for peri-implantitis were examined by Roccuzzo *et al.* They determined that the non-surgical management of peri-implantitis did not yield substantial benefits as a result of the adjunctive application of diode laser. The mean distal/mesial bone levels, biomarker levels and decreases in microbial count at the follow-up visit did not indicate any significant differences among the groups [3]. The short-term efficacy of light-emitting-diode (LED) photo-bio-modulation, which involves multiple sessions of antimicrobial photodynamic therapy (aPDT), was evaluated by Cetiner *et al.* The clinical parameters were not significantly different between the groups, with the exception of gingival recession (GR) [13]. According to Sopi *et al.* the diode laser may be a viable alternative for periodontal treatment, as it offers advantages in both clinical and biochemical parameters [15]. Healing of peri-implant hard and soft tissues may be improved by the explicit application of low-level laser therapy during the postoperative period, as discovered by Palled *et al.* [16]. Talmac *et al.* determined that the Er, Cr:YSGG laser is more effective than the diode laser in the treatment of aggressive periodontitis. IL-37 and IL-1β are cytokines that operate in conjunction and as such, must be assessed in conjunction [17]. low-level laser therapy (LLLT) has demonstrated significant effect in postoperative treatment that targets local bone regeneration and a variety of modalities have been employed to facilitate osseointegration. It has been reported that the administration of LLLT accelerates the process of wound healing by improving it. The impact of photo-bio-modulation is well-documented to be contingent upon a variety of parameters, including wavelength, mode, energy density, exposure duration and treatment frequency [16]. The Treatment of periodontitis has been primarily investigated through the use of photo-bio-modulation and antimicrobial photodynamic therapy, which are mediated by LLL or LED. These methods have shown significant clinical improvements and the elimination of periodontal pathogens. Photo-bio-modulation and antimicrobial photodynamic therapy method have been demonstrated to promote the proliferation and osteo-blastic differentiation of periodontal ligament stem cells at the cellular and molecular levels [13]. Erduran *et al.* evaluated diode laser therapy's immunological and clinical efficacy in treating peri-implantitis as a supplement to non-surgical mechanical therapy. They came to the conclusion that nonsurgical peri-implantitis therapy alone did not produce the same clinical and immunological improvements as the concomitant use of diode lasers [18]. Contrary to our findings, Roccuzzo *et al.* discovered that repeated adjunctive diode laser use in the non-surgical treatment of peri-implantitis did not yield any appreciable advantages when compared to mechanical instrumentation alone [19]. According to Chala *et al.* lasers are an excellent supplementary treatment for peri-implant inflammation [20].

## Conclusion:

The laser groups demonstrated considerable perfection in clinical and biomarker outcomes over time. This shows that both treatment approaches were effective in the treatment of peri-implantitis with 6-month outcomes.

## Figures and Tables

**Table 1:** Comparisons of clinical and biochemical variables of dental implants

**Variables**	**Group I - Control group (CG)**			**p**	**Group II - laser group (LG)**			**p**
	**Baseline**	**3 months**	**6 months**		**Baseline**	**3 months**	**6 months**	
PD (mm)	4.62±3.55	3.64±3.23	3.24±2.24	0	4.64±4.35	3.13±3.64	2.75±2.75	0
PI	2.14±3.54	0.86±3.53	0.42±2.98	0	2.13±3.32	0.54±3.43	0.05±3.23	0
GI	2.05±2.43	2.04±2.32	0.31±2.11	0	0.65±2.23	0.76±2.12	0.06±2.12	0
BOP	87.45±1.54	651.34±	46.34±1.23	0	76.23±	45.23±1.42	25±1.34	0
CAL scores (mm)	5.36±2.23	3.97±2.13	1.35±2.12	0	4.37±2.24	3.16±2.12	0.43±2.13	0
PICF (µl)	2.38±1.234	1.43±1.32	0.97±1.12	0	1.36±1.21	0.64±1.18	0.43±1.08	0
IL-1β (ng/mL)	19.46±1.32	10.24±1.21	6.33±1.13	0	16.35±1.13	10.42±1.32	6.45±1.21	0
PD: Probing Depth
GI: Gingival Index
PI: Plaque Index
PICF: in peri-implant crevicular fluid
IL - interleukine
BOP - bleeding on probing
CAL: clinical attachment level
p - significant
